# Impairment of FOS mRNA Stabilization Following Translation Arrest in Granulocytes from Myelodysplastic Syndrome Patients

**DOI:** 10.1371/journal.pone.0061107

**Published:** 2013-04-12

**Authors:** Xiaomin Feng, Yayoi Shikama, Tsutomu Shichishima, Hideyoshi Noji, Kazuhiko Ikeda, Kazuei Ogawa, Hideo Kimura, Yasuchika Takeishi, Junko Kimura

**Affiliations:** 1 Department of Pharmacology, School of Medicine, Fukushima Medical University, Fukushima, Japan; 2 Department of Cardiology and Hematology, School of Medicine, Fukushima Medical University, Fukushima, Japan; 3 Fukushima Research Institute of Environment and Medicine, Futaba, Japan; 4 Department of Hematology, Kita Fukushima Medical Center, Date, Japan; McGill University, Canada

## Abstract

Although quantitative and qualitative granulocyte defects have been described in myelodysplastic syndromes (MDS), the underlying molecular basis of granulocyte dysfunction in MDS is largely unknown. We recently found that FOS mRNA elevation under translation-inhibiting stimuli was significantly smaller in granulocytes from MDS patients than in healthy individuals. The aim of this study is to clarify the cause of the impaired FOS induction in MDS. We first examined the mechanisms of FOS mRNA elevation using granulocytes from healthy donors cultured with the translation inhibitor emetine. Emetine increased both transcription and mRNA stability of FOS. p38 MAPK inhibition abolished the emetine-induced increase of FOS transcription but did not affect FOS mRNA stabilization. The binding of an AU-rich element (ARE)-binding protein HuR to FOS mRNA containing an ARE in 3′UTR was increased by emetine, and the knockdown of HuR reduced the FOS mRNA stabilizing effect of emetine. We next compared the emetine-induced transcription and mRNA stabilization of FOS between MDS patients and healthy controls. Increased rates of FOS transcription by emetine were similar in MDS and controls. In the absence of emetine, FOS mRNA decayed to nearly 17% of initial levels in 45 min in both groups. In the presence of emetine, however, 76.7±19.8% of FOS mRNA remained after 45 min in healthy controls, versus 37.9±25.5% in MDS (P<0.01). To our knowledge, this is the first report demonstrating attenuation of stress-induced FOS mRNA stabilization in MDS granulocytes.

## Introduction

Myelodysplastic syndromes (MDS) are clonal disorders of hematopoietic stem cells characterized by ineffective hematopoiesis in one or more lineages of blood cells [1]. Terminally differentiated granulocytes in MDS patients exhibit numerical reduction which is thought to result from increased apoptosis of progenitors in bone marrow [2], and functional abnormalities such as reduction of bactericidal and fungicidal activities, impaired production of reactive oxygen species, and aberrant migration ability [3]–[9]. However, molecular basis of granulocyte defects is largely unknown.

FOS, a component of a transcription factor activator protein 1 (AP-1) [10], is a pivotal regulator of apoptosis and production of inflammatory mediators in granulocytes [11]–[14]. A DNA microarray analysis identified FOS as one of the seven most downregulated genes in quiescent granulocytes derived from MDS patients [15]. Since splicing isoforms with a premature termination codon (PTC) of various molecules were found in MDS by us [16] and others [17], [18] and mRNAs harboring a PTC are degraded by a “nonsense-mediated mRNA decay (NMD)” surveillance system [19] that can be inhibited by translation inhibitors [20], we hypothesized that excessive production of NMD-sensitive mRNA resulted in their aberrantly low expression, causing cellular dysfunction in MDS. To examine this hypothesis, we previously investigated whether cessation of NMD by the translation inhibitor puromycin unveiled excessive production of NMD-sensitive mRNA of downregulated genes in MDS granulocytes and restored expression [21]. Unexpectedly, FOS mRNA with no PTC was time-dependently increased in healthy granulocytes, and the increase rate was significantly lower in MDS than in controls at any time points tested. This suggested that not only basal FOS mRNA levels but also regulatory processes of FOS expression under environmental stimuli were impaired in MDS. FOS is also known as one of the immediate early genes (IEGs), whose expression levels are low in quiescent cells but rapidly increase in response to a broad range of stimuli from proliferative factors to various types of cellular stress [22]. Eukaryotic cells exposed to genotoxic stress inhibit general translational activities by phosphorylation of translation initiation factor eIF2α and stabilize labile mRNAs [23], [24]. FOS mRNA elevation following translation inhibition can be regarded as an aspect of stress response, and the attenuated FOS induction in MDS may imply impairment in the process of stress response of MDS granulocytes. If so, the impairment behind aberrant FOS mRNA induction influences other IEGs, which may affect granulocyte functions and granulopoiesis.

To date, both *transcriptional* and *post-transcriptional* regulations of FOS expression have been reported. Transcriptional regulation seems to involve MAPK signaling pathway. In a human osteosarcoma cell line, the oxidant arsenite and the translation inhibitor anisomycin promoted FOS transcription within 30 min via three MAPKs, p38, extracellular signal-regulated kinases (ERK), and c-Jun N-terminal kinase (JNK) [25]. Lipopolysaccharide (LPS)-induced FOS transcription in rat glial cells was mediated by p38 MAPK but not ERK [26]. On the other hand, post-transcriptional regulation of FOS mRNA requires its 3′UTR. The removal of 3′UTR resulted in prolongation of FOS mRNA turnover [27], and recent studies identified AU-rich element (ARE) within 3′UTR of many IEGs including FOS, to which a number of mRNA stability-regulating proteins and miRNAs [28]–[31] bind. ARE in FOS mRNA 3′UTR was shown to bind to Hu antigen R (HuR) and AUF1 [32]. HuR, a member of the ELAV/Hu family, is ubiquitously expressed and its binding to ARE-containing mRNAs in the cytoplasm generally leads to mRNA stabilization [33]–[35]. AUF1, which belongs to the family of AUF1/hnRNPD RNPs, is expressed as four alternatively spliced isoforms designated by their molecular weights as p37, p41, p42, and p45 [36], [37]. The binding of AUF1 generally results in mRNA destabilization, while mRNA stabilizing effects of some AUF1 isoforms were reported in a few cell types [36], [38]–[40].

This study aimed to clarify the cause of the aberrant FOS mRNA induction following translation arrest in granulocytes from MDS patients. We examined whether the attenuated FOS mRNA elevation in MDS was exclusive to translation-inhibiting stimuli, and demonstrated the mechanism of FOS induction under translation inhibition in granulocytes, and identified the impaired process of FOS induction in MDS.

## Materials and Methods

### Reagents

Emetine, lipopolysaccharides (LPS), 5, 6-Dichlorobenzimidazole 1-β-D-ribofuranoside (DRB), vanadyl ribonucleoside complexes, protein G sepharose 4B beads and rabbit polyclonal anti-Actin (a2066) were purchased form Sigma-Aldrich (St. Louis, MO, USA), and human granulocyte-macrophage colony-stimulating factor, (GM-CSF) was from Pepro Tech (Rocky Hill, NJ, USA). Streptavidin T1 magnetic beads, streptavidin C1 magnetic beads, Moloney murine leukemia virus reverse transcriptase, Click-iT Nascent RNA Capture Kit, and RNaseOUT were products of Life Technologies (Carlsbad, CA, USA). SB203580, SP600125, proteinase inhibitor cocktail were purchased from Calbiochem (Darmstadt, Germany), and U0126 was from Promega (Madison, WI, USA). T7 RNA polymerase, Biotin RNA Labeling Mix and tRNA were obtained from Roche Applied Science (Mannheim, Germany), and random hexamers and SYBR premix Ex Taq were from TaKaRa Bio (Otsu, Japan). Mouse monoclonal anti-HuR (3A2), horse radish peroxidases (HRP)-conjugated anti-mouse (sc-2005) and rabbit (sc-2004) IgG were obtained from Santa Cruz Biotechnology (Santa Cruz, CA, USA). Rabbit polyclonal anti-hnRNP D/AUF1 (ab61193) was a product of Abcam (Cambridge, UK).

### Blood donors

Peripheral blood was obtained from twenty-four patients with MDS and seventeen age-matched healthy controls. Specific diagnoses included three of refractory cytopenia with unilineage dysplasia (RCUD), one of refractory anemia (RA) with ringed sideroblasts (RARS), fourteen of refractory cytopenia with multilineage dysplasia (RCMD), and two of RA with excess blasts-1 (RAEB-1) according to WHO 2008 criteria [41]. Hematological and clinical findings of the patients are summarized in Table 1.

**Table 1:** Hematological and Clinical Findings of Patients.

No	Age/Sex	subtype	WBC (×10^9^/L)	Neutrophils (×10^9^/L)	Hb (g/dL)	PLT (×10^10^/L)	Cytogenetics	Therapy
1	74/M	RCMD	2.7	1.4	10.2	8.7	46,XY	none
2	74/F	RCUD	2.1	1.3	12.1	13.8	46,XX	none
3	88/M	RCUD	3.5	1.6	11.8	17.1	46,XY	transfusion (RBC)
4	67/M	RAEB-1	1.4	0.6	12.6	6.8	47,XY,+8add(11)(q21)	G-CSF, transfusion (RBC, PLT)
5	68/F	RCMD	5.5	2.0	13.0	1.9	47,XX,+9	PSL
6	67/M	RCMD	5.2	1.9	8.7	12.4	46,XY	CyA, androgen, V.D+V.K
7	70/M	RCMD	5.3	1.6	9.6	1.0	46,XY	CyA, insulin
8	67/M	RAEB-1	3.8	1.9	7.6	21.9	46,XY 46,XY,del(20)(q12q13)	transfusion (RBC)
9	39/F	RCMD	3.5	1.5	6.2	0.9	46,XX	transfusion (PLT), PSL
10	55/M	RCMD	2.2	1.0	5.8	3.2	46,XY	transfusion (RBC)
11	66/M	RCMD	1.4	0.4	10.4	19.9	46,XY	Transfusion (RBC)
12	67/M	RCUD	4.7	2.5	11.4	17.7	47,XY add(8)(q11.1),+add(8)	none
13	61/F	RCMD	2.9	1.0	7.4	1.8	46,XX,i(18)(q10) 46XX,i(18)(q10),i(18)(q10)	G-CSF, transfusion (RBC, PLT), CyA, ATG
14	88/M	RCMD	3.0	1.0	5.7	39.5	46,XY 45,X,-Y,+1,der(1;16)(q10;q10)	EPO, transfusion(RBC)
15	75/M	RCMD	5.7	3.9	6.2	1.6	46,XY	transfusion (RBC, PLT)
16	87/F	RCMD	3.9	2.9	9.8	1.5	47,XX,+8	transfusion (PLT)
17	68/F	RCMD	2.2	0.7	9.9	19.4	46,XX	CyA
18	78/M	RCMD	1.3	0.4	6.9	8.3	46,XY	transfusion (RBC, PLT)
19	69/F	RARS	4.8	2.6	6.7	20.9	46,XX	transfusion (RBC)
20	88/F	RCMD	1.2	0.4	7.5	4.9	47,XY, +8	transfusion (RBC, PLT)
21	80/F	RCUD	3.0	1.5	7.0	33.0	46XX	transfusion (RBC)
22	80/M	RCUD	7.7	6.1	8.0	33.3	46XY	transfusion (RBC)
23	71/M	RARS	2.9	2.2	8.0	33.9	46XY	transfusion (RBC)
24	86/M	RAEB-1	3.8	1.6	9.5	14.8	46XY	transfusion (RBC)

M, male; F, female; WBC, white blood cells; Hb, hemoglobin concentration; PLT, platelets; RBC, red blood cells; G-CSF, granulocyte colony-stimulating factor; PSL, prednisolone; CyA, cyclosporin A; V.D, Vitamin D; V.K, Vitamin K; ATG, antithymocyte globulin; EPO, erythropoietin.

### Ethics

This study, and the process of securing informed consent from patients and healthy controls, were approved by the Ethics Committee of Fukushima Medical University (approval number: 804), which is guided by local policy, national laws, and the World Medical Association Declaration of Helsinki. All study participants provided their written consent.

### Cells

As previously described [42], the granulocyte fraction was obtained from peripheral blood by centrifugation through Lymphoprep (l.077 g/mL, Axis-Shield, Oslo, Norway) followed by hypotonic lysis of erythrocytes. Staining of the fractionated cells with May-Grünwald and Giemsa solutions revealed that more than 90% of the cells were neutrophilic granulocytes. A human promyelocytic leukemia cell line HL60 was purchased from Riken BRC Cell Bank (Tsukuba, Japan). The granulocytes and HL60 cells were cultured in RPMI 1640 (Wako, Mie, Japan) supplemented with 10% (v/v) heat-inactivated fetal bovine serum (FBS) (Nichirei biosciences, Tokyo, Japan) at a concentration of 5×10^6^ and 1×10^6^ cells/mL, respectively, for stimulation with FOS inducers.

### RNA extraction, reverse transcription, and real-time PCR

Total cellular RNA was extracted from granulocytes and HL60 cells using RNeasy Plus Mini Kit (Qiagen, Hilden, Germany) and ISOGEN (NIPPON GENE, Toyama, Japan), respectively. First-strand cDNA was synthesized as described previously [43]. The primer sequences are shown in Table 2.

**Table 2:** Primers used for PCR.

No.	Primer	Sequence (5′→3′)
1	FOS, forward	GGGATAGCCTCTCTTACTACCACT
2	FOS, reverse	CCTCCTGTCATGGTCTTCACAAC
3	β-actin, forward	CAAGAGATGGCCACGGCTGCT
4	β-actin reverse	TCCTTCTGCATCCTGTCGGCA
5	FOS 3′UTR for RIP, forward	GGGAGGACCTTATCTGTGCGTGAA
6	FOS 3′UTR for RIP, reverse	GGGAACAATACACACTCCATGCGT
7	GAPDH 3′UTR for RIP forward	TGCACCACCAACTGCTTAGC
8	GAPDH 3′UTR for RIP reverse	GGCATGGACTGTGGTCATGAG
9	T7-FOS for biotinylation forward	CCAAGCTTCTAATACGACTCACTATAGGGAGAGAGGACCTTATCTGTGCGTG
10	FOS for biotinylation reverse	CACAAAAGCTGTTACACAGCGGT
11	T7-GAPDH for biotinylation forward	CCAAGCTTCTAATACGACTCACTATAGGGAGACCTCAACGACCACTTTGTCA
12	GAPDH for biotinylation reverse	GGTTGAGCACAGGGTACTTTATT
13	FOS for sequencing, forward 1	ATCTGGGTCCTTCTATGCAG
14	FOS for sequencing forward 2	CTGTGTTCCTGGCAATAGTGTG
15	FOS for sequencing, reverse	CCACATGTCAAAAGACCTCAAGG

### Detection of newly synthesized RNA

Newly synthesized RNA was isolated using Click-iT Nascent RNA Capture Kit according to the manufacturer's protocol. Briefly, granulocytes were cultured with 5-Ethynyl Uridine (EU), and after the first 30 min, emetine was added. For the analysis of mRNA decay, EU was washed out with RPMI1640 supplemented with 10% FBS twice prior to emetine stimulation. Total cellular RNA extracted from the EU-treated cells was incubated with biotin azide for 30 min at room temperature (RT) and stored at −70°C overnight. The biotinylated RNA was collected by Streptavidin T1 Magnetic Beads and subjected to real-time RT-PCR.

### siRNA transfections

Fifty nM of siRNA (Ambion, Austin, TX, USA) targeting human HuR (sense GCGUUUAUCCGGUUUGACAtt and antisense UGUCAAACCGGAUAACGCaa) or control siRNA (Ambion) were introduced into 2.4×10^6^ HL60 cells suspended in 800 µL of Gene Pulser Electroporation Buffer Reagent (Bio-Rad Laboratories, Hercules, CA, USA) by square-pulse electroporation (280 V, 12 msec) using a Gene Pulser (Bio-Rad). After 42 hours, cells were treated with the indicated drugs.

### Cell lysate preparation

Cytoplasmic and nuclear lysates were prepared as previously described [44]. To prepare total cell lysates from granulocytes, the cells were precipitated in 10% trichloroacetic acid (Wako) for 30 min on ice. The TCA-precipitated fraction was treated with a lysis solution containing 9 M urea, 2% Triton X-100, 1 mM DTT and disrupted by ultrasonication, followed by an addition of 2% lithium dodecyl sulfate and further ultrasonication. HL60 lysate was prepared using 1% Nonidet P-40 (NP-40) Buffer.

### Immunoblotting

Proteins were separated by electrophoresis on a 12% polyacrylamide gel, and transferred onto Immobilon-P Transfer membranes (Millipore, Billerica, MA, USA) using a semi-dry transfer apparatus. After blocking with 5% milk dissolved in TBS-T (10 mM Tris, pH8.0, 150 mM NaCl, 0.5% Tween 20), the membrane was incubated with primary antibodies at RT for 1 hour followed by incubation with HRP-conjugated secondary antibodies for 1 hour at RT. Signals were detected by ECL Western Blotting Detection Reagents (GE Healthcare, Buckinghamshire, UK).

### RNP complex immunoprecipitation (RIP)

RIP assay was performed as previously described [45] with minor modifications. Briefly, granulocytes were fixed by 1% formaldehyde in PBS, quenched by 0.25 M glycine [pH 7.0], and lysed by RIPA buffer (50 mM Tris-HCl [pH7.5], 1% NP-40, 0.5% sodium deoxycholate, 0.5% SDS, 1 mM EDTA, 150 mM NaCl) containing a 1% protease inhibitor cocktail. After three rounds of 20-second sonication and centrifugation, the supernatant was incubated with antibody-coated Protein G Sepharose 4B beads in the presence of 400 µU/mL RNase OUT, 25 µg/mL tRNA and 5% vanadyl ribonucleoside complexes solution for 2 h at 4°C. The bead-attached RNA was extracted and subjected to quantitation of 3′UTR of FOS and GAPDH by real-time RT-PCR. The precipitated proteins were analyzed by Western blotting.

### Biotin pulldown assay

To obtain the biotinylated mRNA 3′UTR of FOS and GAPDH, the templates for *in vitro* transcription were synthesized by PCR amplification using forward primers that contained T7 RNA polymerase promoter sequence at their 5′ends (Table 2) and cDNA from healthy granulocytes as a template. The templates were transcribed using T7 RNA polymerase and Biotin RNA Labeling Mix. Biotin pulldown assay was carried out by incubating cytoplasmic or nuclear lysates with biotinylated transcripts in a 10∶1 or 40∶1 ratio, respectively, for 1 hour at RT in the presence of 400 mU/mL RNase OUT, 25 µg/mL tRNA and 5% vanadyl ribonucleoside complex. RNA was collected by Streptavidin C1 Magnetic Beads and proteins bound to RNA were analyzed by Western blotting.

### Sequencing

PCR products generated from patient-derived cDNA by primers no. 13 and 15 in Table 2 were sequenced on an ABI PRISM 3130 x1 genetic analyzer (Applied Biosystems, Carlsbad, CA, USA).

### Statistical analysis

Comparison between the two groups was performed by the Mann-Whitney test or the paired *t* test. Data from more than three groups were compared using ANOVA (IBM SPSS Statistics, 17.0). *P* values less than 0.05 were considered significant.

## Results

### Emetine but not GM-CSF or LPS induced aberrant FOS mRNA elevation in MDS granulocytes

**Figure 1:**
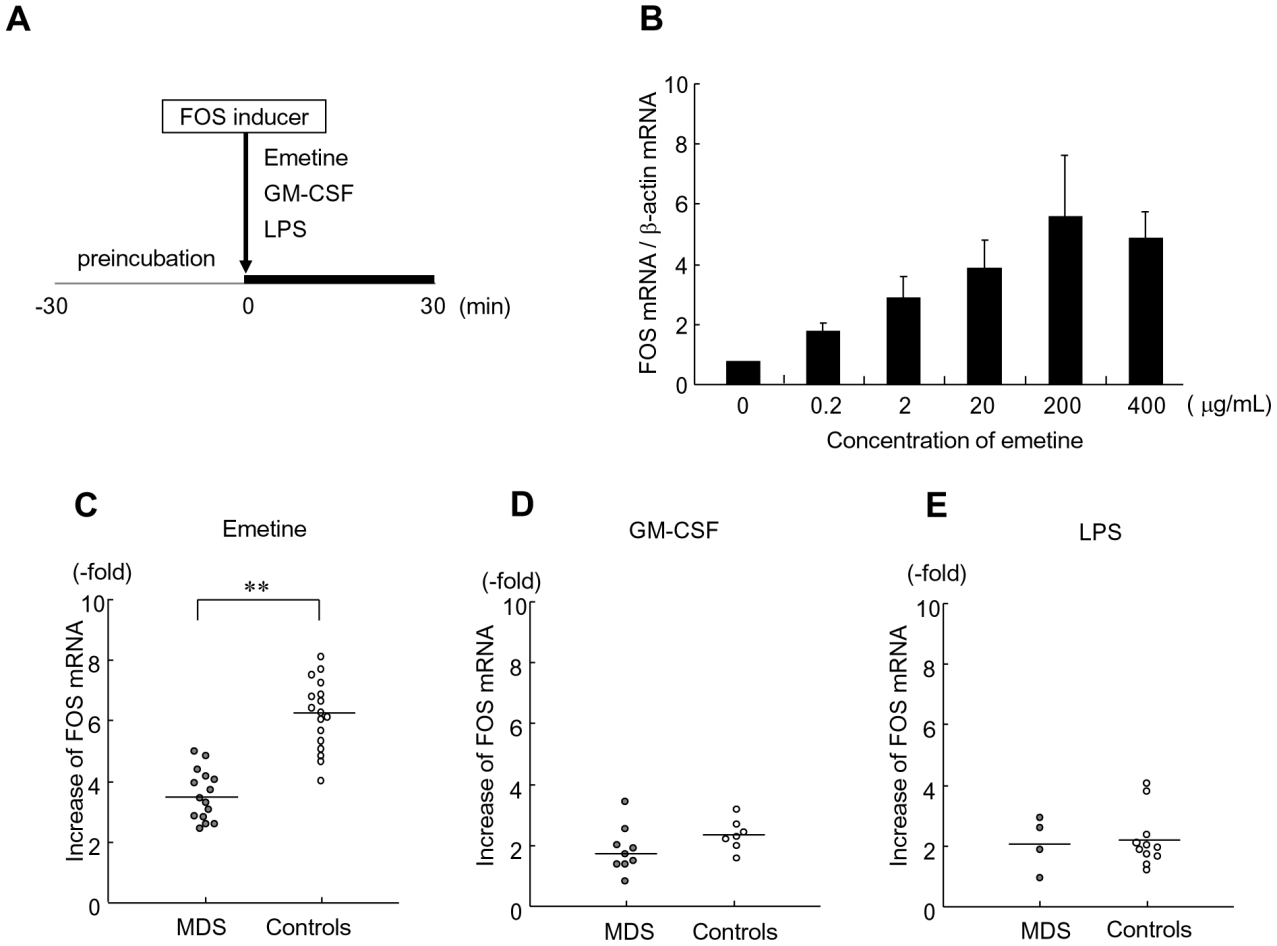
FOS mRNA elevation by various stimuli in human granulocytes. (A) Experimental design. After 30-min preincubation, granulocytes were cultured with an indicated FOS inducer for another 30 min. (B) Dose response to emetine. Granulocytes isolated from healthy volunteers were cultured with the indicated concentrations of emetine. Expression levels of FOS mRNA were normalized by those of an internal control β-actin mRNA. The normalized value in the absence of emetine was set as 1.0. Mean ± SD of three to seven experiments are presented. (C)(D)(E) Comparison of FOS mRNA elevation between MDS patients and healthy controls. Granulocytes were cultured with 200 µg/mL emetine (C), 5 ng/mL GM-CSF (D), or 100 ng/mL LPS (E). The fold increase of the ratios of FOS mRNA to β-actin mRNA by a FOS inducer was plotted, and the horizontal bars represent the means. The statistical comparison was performed by the Mann-Whitney test. **P<0.01.

To examine whether puromycin exclusively impaired FOS mRNA elevation in MDS, we analyzed the effect of the translation inhibitors emetine and cycloheximide in granulocytes from healthy donors (Figure 1A). Emetine (Figure 1B), as well as puromycin and cycloheximide (data not shown), increased FOS mRNA levels in a dose-dependent manner. We decided to use 200 µg/mL of emetine for the comparison of FOS mRNA elevation between MDS and healthy granulocytes, because this concentration consistently provided maximum effect. Patients showed a significantly attenuated increase of FOS mRNA (3.5±0.8-fold) compared to that of the controls (6.2±1.1-fold) (*P*<0.01) (Figure 1C).

The effects of FOS inducers other than translation inhibitors were also examined. When granulocytes were cultured with GM-CSF instead of emetine, FOS mRNA elevation did not significantly differ between the controls (2.3±0.5-fold) and MDS patients (1.9±0.8-fold) (Figure 1D). LPS also showed similar FOS effects in the controls (2.2±0.9-fold) and MDS (2.4±1.1-fold) (Figure 1E).

### Both MAPK p38-dependent and MAPK-independent processes are involved in FOS mRNA elevation by emetine in granulocytes

**Figure 2:**
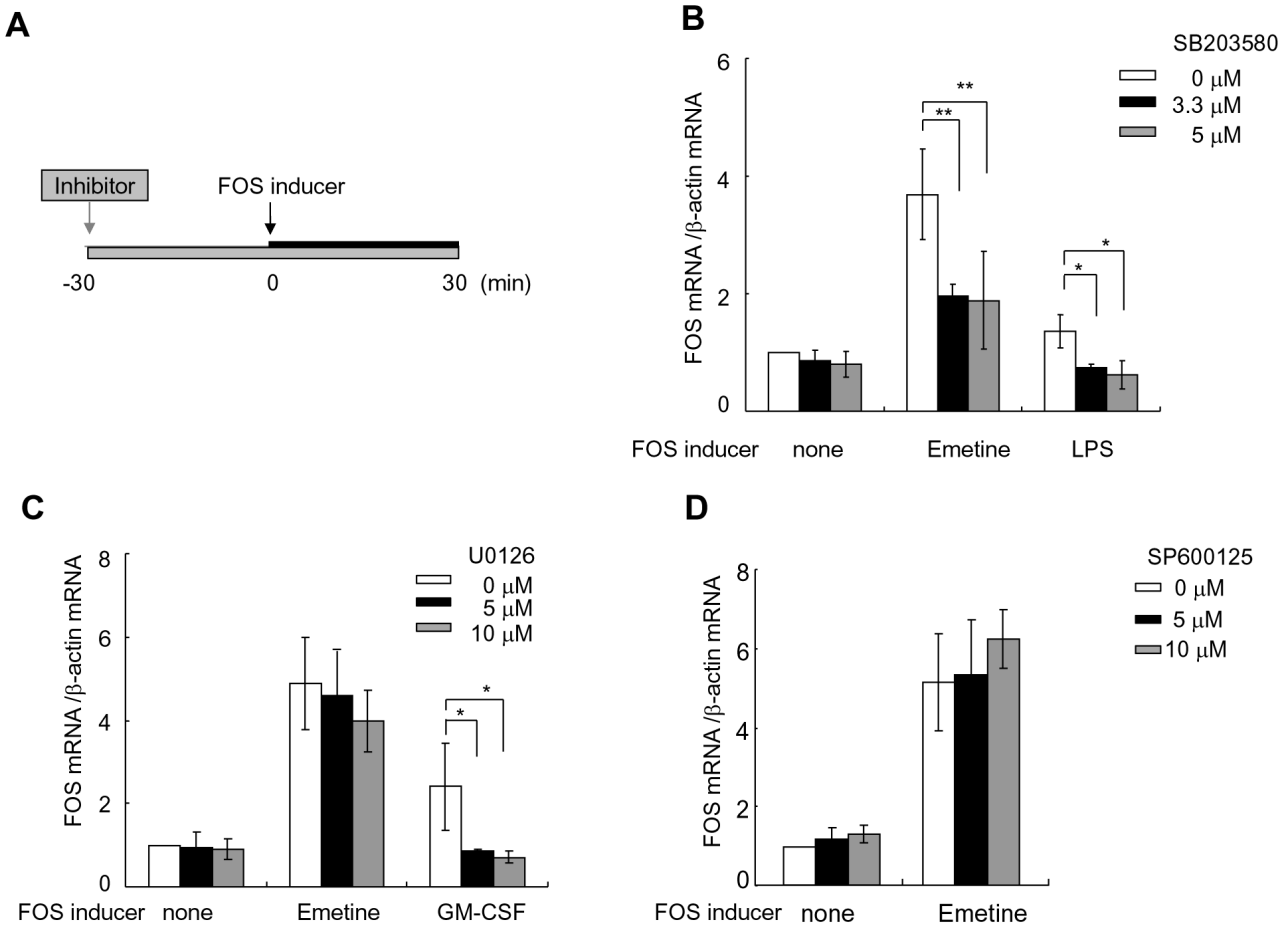
Effects of MAPK inhibitors. (A) Experimental design. Granulocytes isolated from healthy individuals were cultured in the presence of a MAPK inhibitor for a total of 60 min. After the initial 30 min, a FOS inducer was added. (B)(C)(D) Effects of MAPK inhibition. A p38 MAPK inhibitor SB203580 (B), an ERK inhibitor U0126 (C), or a JNK inhibitor SP600125 (D) was used. The ratio of FOS mRNA to β-actin mRNA in untreated cells was assigned for 1.0. The results shown are mean ± SD of three to seven experiments. The values were statistically compared by ANOVA. *P<0.05, **P<0.01.

The involvement of MAPKs in emetine-induced FOS mRNA elevation in healthy granulocytes was examined using inhibitors for three kinds of MAPKs (Figure 2A). In the presence of 3.3 µM MAPK p38 inhibitor SB203580, FOS mRNA elevation by LPS was completely blocked (0.7±0.1-fold elevation), while emetine-induced FOS mRNA elevation was reduced only by 34.9±10.0% and further reduction was not observed with a higher concentration of the inhibitor (Figure 2B). Five µM of MEK1/2 inhibitor U0126, which was sufficient to cease FOS mRNA elevation by GM-CSF, did not alter the effects of emetine (Figure 2C). Five and 10 µM of JNK inhibitor SP600125 did not affect FOS mRNA elevation by emetine (Figure 2D). These results indicated that both p38-dependent and MAPK-independent processes were involved in FOS mRNA elevation by emetine in granulocytes.

### Emetine increases transcription via MAPK p38

**Figure 3:**
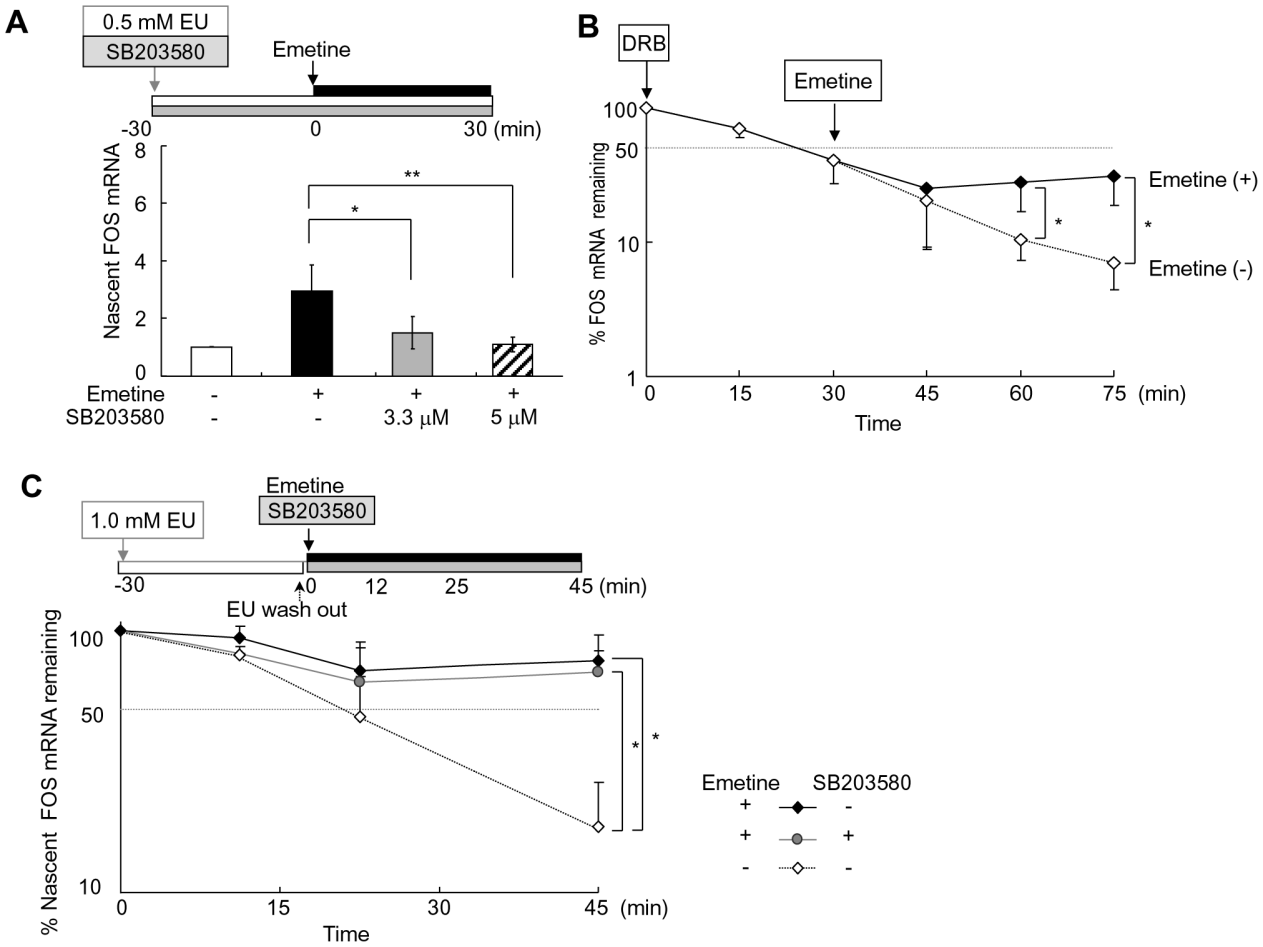
Effects of emetine on FOS mRNA synthesis and decay. (A) Effects of emetine on FOS transcription. Healthy granulocytes were cultured in the presence of 0.5 mM ethynyl uridine (EU) for a total of 60 min with or without a p38 inhibitor SB203580, and after the initial 30 min, emetine was added. The ratio of EU-incorporated nascent FOS mRNA to nascent β-actin mRNA in untreated cells was set as 1.0, and the mean ± SD of thirteen experiments with emetine alone, and eight experiments with emetine and SB203580 were presented. *P<0.05, **P<0.01 by ANOVA. (B) Effects of emetine on FOS mRNA decay. Transcription was inhibited by 25 µg/mL of DRB, and emetine was added to culture medium after the initial 30 min. The cells were harvested for FOS mRNA quantification every 15 min. The mean ± SD of four experiments were shown. *P<0.05 by the Mann-Whitney test. (C) Effects of MAPK p38 inhibition on nascent FOS mRNA decay. Following the incubation of granulocytes with 1.0 mM EU for 30 min, EU was washed out by RPMI 1640 twice, and the cells were treated with emetine in the presence and absence of SB203580 for a further 45 min. The ratio of EU-incorporated nascent FOS mRNA to β-actin mRNA at 0 min was assigned for 100%. The graph shows mean ± SD of six experiments. **P<0.01 by ANOVA.

To clarify the roles of p38 MAPK pathway in FOS mRNA elevation by emetine, we first examined the effects of emetine on FOS transcription in the absence or presence of a p38 inhibitor, using healthy granulocytes. As shown in Figure 3A, emetine increased nascent FOS mRNA 2.8±0.8-fold. When 3.3 and 5 µM of SB203580 were added, the increased rate of nascent FOS mRNA fell to 1.5±0.5-fold (*P*<0.05) and 1.1±0.3-fold (*P*<0.01), respectively.

### Emetine stabilizes FOS mRNA p38 independently

We next examined the effects of emetine on FOS mRNA decay and the involvement of p38 MAPK. In the presence of a transcription inhibitor DRB, FOS mRNA decreased to 9.6±4.4% and 6.4±2.8% of the initial level at 60 and 75 min, respectively (Figure 3B). When emetine was added after the first 30 min, 27.5±17.5% and 32.7±18.5% of FOS mRNA remained at 60 and 75 min, respectively. This FOS mRNA-stabilizing effect of emetine was confirmed by analyzing the decay of EU-labeled nascent FOS mRNA (Figure 3C). Nascent FOS mRNA decreased to 19.9±10.0% of the initial level in 45 min in the absence of emetine. In the emetine-treated cells, 76.1±20.4% of nascent FOS mRNA remained, which was not significantly altered by the addition of 5 µM of SB203580 (69.6±15.4%), suggesting that emetine stabilized FOS mRNA via MAPK-independent mechanisms.

### Emetine increases binding of HuR to FOS mRNA 3′UTR in granulocytes

**Figure 4:**
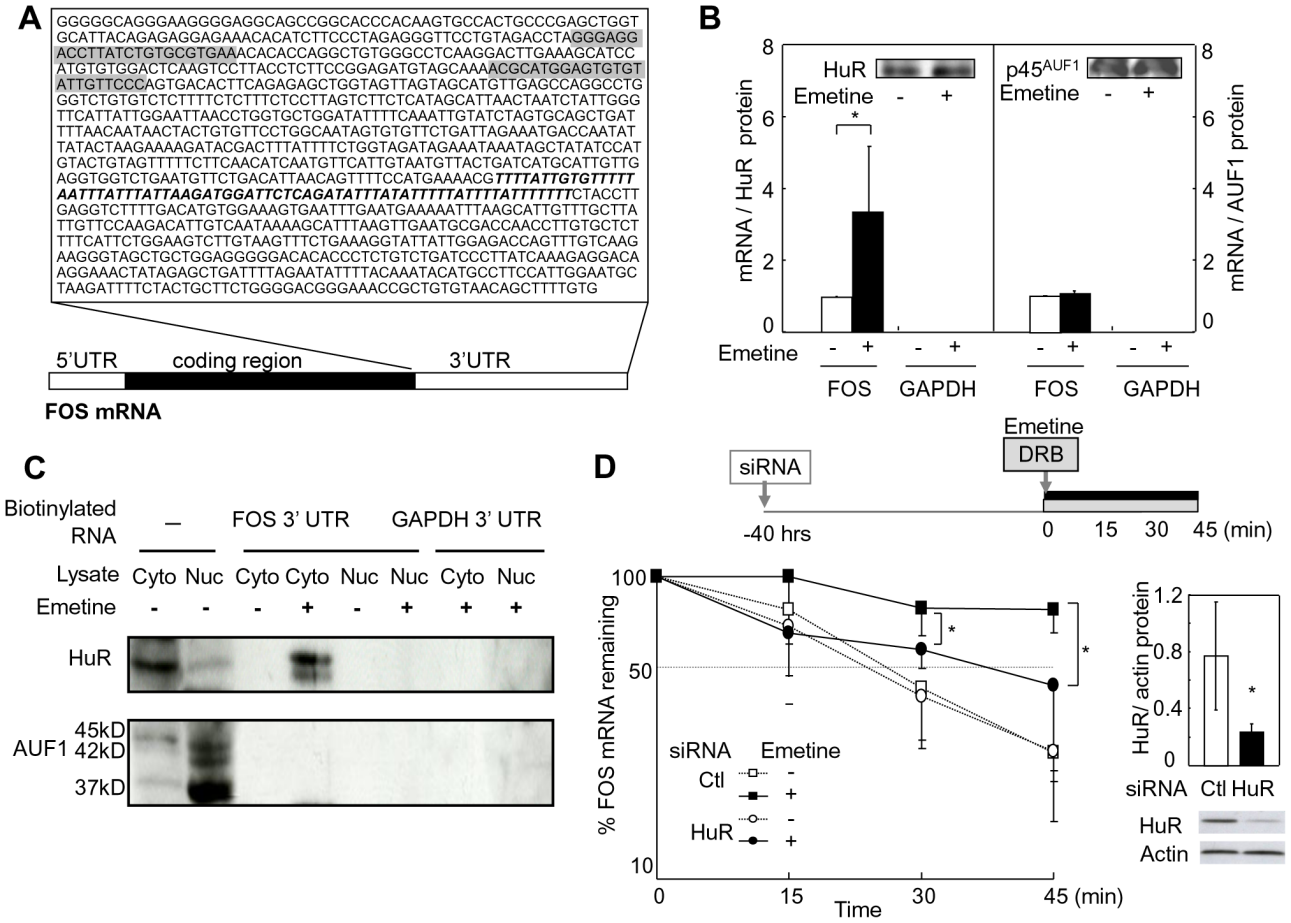
Involvement of HuR and AUF1 on FOS mRNA stabilization by emetine. (A) Location of PCR primers for RIP assay and sequence of pull-down assay probe. The sequence of biotinylated transcript for pull-down assay is in the square. The forward and reverse PCR primers used for RIP assay are highlighted in gray. The bold italic letters indicate the binding site for HuR. (B) RIP assay with anti-HuR and anti-AUF1. The whole cell lysates of emetine-treated and untreated granulocytes were subjected to immunoprecipitation of HuR and AUF1. FOS and GAPDH mRNAs contained in the precipitants were quantified by real-time RT-PCR and normalized by precipitated protein amounts. The values in untreated cells, which is shown by white bars, were assigned for 1.0. Black bars present the data from emetine-treated cells. *P<0.05 by paired *t* test. (C) Pull-down assay with biotinylated RNA. Cytoplasmic (Cyto) and nuclear (Nuc) lysates were incubated with biotinylated transcripts of FOS or GAPDH, and HuR and AUF1 bound to the transcripts were detected by immunoblotting. The left two lanes show the lysates without pull down as positive controls. (D) Effects of HuR knockdown on FOS mRNA stabilization by emetine. Control or HuR-directed siRNAs were introduced to HL60 by electroporation. After 40 hours, HuR protein levels were examined (right panel), and the cells were cultured in the presence of DRB with or without emetine for 45 min for the analysis of the remaining FOS mRNA levels (left panel). Each point presents mean value of four to seven independent experiments. Error bars indicate SD. *P<0.05 by the Mann-Whitney test.

To examine whether ARE-binding proteins HuR and AUF1 contributed to FOS mRNA stabilization by emetine, HuR and AUF1 were immunoprecipitated under conditions that preserved RNP integrity. Emetine increased FOS mRNA 3′UTR (Figure 4A) co-precipitated with HuR 3.1±1.6-fold, while GAPDH mRNA 3′UTR, which had no ARE, was not detected regardless of emetine treatment (Figure 4B). In contrast, the amounts of FOS mRNA 3′UTR contained in the AUF1 precipitants, which was mainly p45 isoform, did not differ between untreated and emetine-treated cells (Figure 4B). The binding of FOS mRNA to ARE-binding proteins was further analyzed by pull-down assay using biotinylated transcripts (Figure 4A). HuR formed a complex with biotinylated FOS mRNA 3′UTR in the cytoplasm derived from emetine-treated cells (Figure 4C). Biotinylated GAPDH 3′ UTR transcripts served as a negative control. AUF1 was not detected in either untreated or emetine-treated cells.

### HuR stabilizes FOS mRNA in emetine-treated cells

The functional consequence of increased binding of HuR to FOS mRNA was studied by RNA interference of HuR expression in HL60 cells, which were used instead of short-lived neutrophilic granulocytes. HuR protein levels were decreased by 70% in HuR siRNA-treated cells (Figure 4). Without emetine, FOS mRNA decayed to similar levels in the cells treated with HuR siRNA (26.5±5.6%) and control siRNA (26.2±10.7%) in 45 min. In the presence of emetine, however, the remaining FOS mRNA at 30 and 45 min was significantly lower in HuR siRNA-treated cells (57.3±7.7% and 43.8±20.1%, respectively) than in control cells (78.3±14.7%, *P*<0.05 and 77.4±12.8%, *P*<0.05), suggesting that emetine stabilized FOS mRNA via HuR (Figure 4D).

### FOS mRNA stabilizing process by emetine is impaired in MDS granulocytes

**Figure 5:**
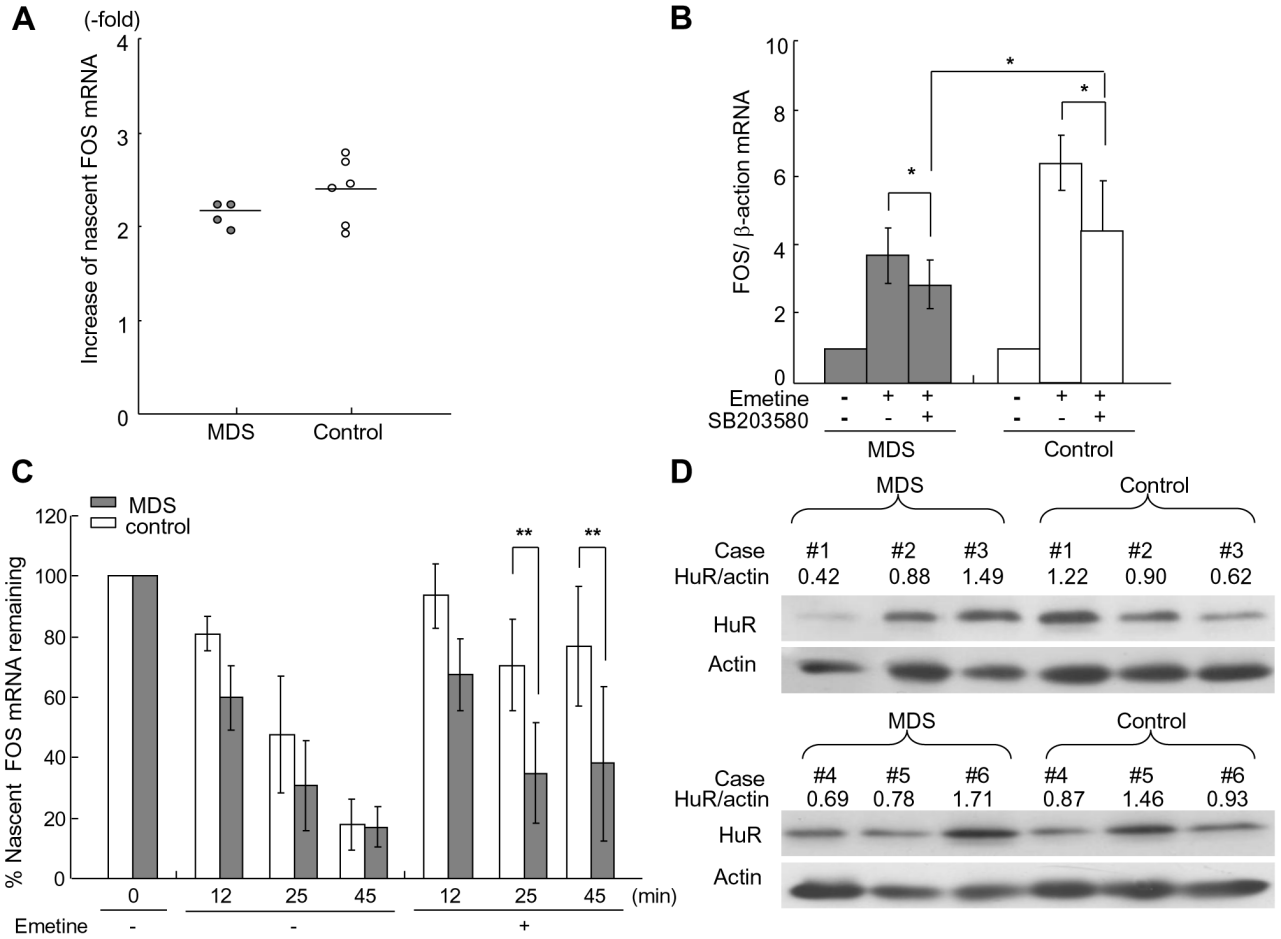
Comparison of FOS transcription and mRNA stabilization by emetine between MDS patients and the healthy controls. (A) FOS transcription by emetine. Granulocytes were treated as shown in Figure 3A. The increase in the ratios of EU-incorporated nascent FOS to β-actin mRNA was plotted. The bars represent the mean values. (B) FOS mRNA elevation by emetine in the presence of MPAK p38 inhibitor. Granulocytes from seven patients and nine controls were treated as in Figure 2A with 5 µM SB203580. The values in untreated cells were set as 1.0. Gray and white bars indicate MDS and control, respectively. *P<0.05 by ANOVA. (C) Nascent FOS mRNA remaining for 45 min. The decay of the nascent mRNA labeled with EU was compared between MDS and the controls at 12, 25, and 45 min. The mean ± SD of nascent FOS mRNA levels normalized by β-actin were presented. **P<0.01 by the Mann-Whitney test. (D) Comparison of HuR protein expression between MDS and the controls by western blotting. The numbers above the photos indicate the ratio of HuR band intensity to actin band.

We finally attempted to determine which process was impaired in MDS granulocytes, p38-mediated FOS transcription or HuR-mediated FOS mRNA stabilization. As shown in Figure 5A, the increase of nascent FOS mRNA by emetine treatment for 30 min was 2.2±0.1-fold in MDS granulocytes, which was not significantly different from that in the control cells (2.4±0.3-fold). To compare the FOS mRNA stabilizing effects of emetine between MDS patient- and healthy volunteer-derived cells, emetine-induced FOS transcription was blocked by MAPK p38 inhibition. As shown in Figure 5B, p38 inhibitor reduced FOS mRNA elevation by emetine by 30.4±15.5% and 38.0±22.5% in the cells from MDS patients and the controls, respectively. As a consequence, FOS mRNA increase by emetine remained significantly smaller in MDS patients (2.9±0.7-fold) than in controls (4.4±1.5-fold, *P*<0.05), suggesting that the mRNA stabilizing process was impaired in MDS granulocytes. To directly confirm this result, decay of nascent FOS mRNA was analyzed in MDS and the controls (Figure 5C). In the absence of emetine, nascent FOS mRNA levels decreased to 59.9±10.6%, 30.8±14.8% and 17.0±6.6% after 12, 25 and 45 min in MDS granulocytes, respectively. The differences from remaining FOS mRNA in controls (81.0±5.8% at 12 min, 47.6±19.5% at 25 min, and 17.8±8.5% at 45 min) were not statistically significant at any time points. In contrast, in the presence of emetine, MDS showed significantly lower FOS mRNA levels remaining at 25 and 45 min (34.8±16.7% and 37.9±25.5%, respectively) than the healthy controls (70.6±15.1%, *P*<0.01 and 76.7±19.8%, *P*<0.01).

### HuR protein was reduced in an MDS patient

Sequencing of FOS mRNA 3′UTR between the stop codon and 3′end of the HuR binding domain did not reveal any mutations in 13 patients tested. To examine the possibility that reduced HuR expression in MDS is responsible for the impaired FOS mRNA stabilization, HuR protein levels in six patients and six controls were analyzed by Western blotting. Compared with controls whose ratio of HuR to actin was 1.00±0.29, one patient showed a reduced level of HuR protein with the ratio of 0.42, and other patients preserved HuR protein levels similar to those of the controls with the mean ratio of 1.00 (Figure 5D).

## Discussion

This study demonstrated that (1) translation inhibitor emetine increased FOS transcription via MAPK p38 signaling pathway and FOS mRNA stability via interaction with HuR in granulocytes and (2) the aberrant elevation of FOS mRNA in MDS patients resulted from impaired stabilization process.

The aberrant FOS induction in MDS granulocytes was exclusive to translation-inhibiting stimuli, because it was reproduced by another translation inhibitor emetine in addition to puromycin [21] but not by other FOS inducers GM-CSF and LPS. Emetine that stabilizes polysomes, as well as puromycin that dismantles polysomes, has been used for analyses of intracellular events under environmental stress, because eukaryotic cells exposed to stress inhibit general translation activity and construct stress granules (SG) and processing bodies (PB) where selected mRNAs are stabilized [23], [24]. Although some differences have been shown between the effects of emetine and puromycin on formation of SGs and PBs, translational arrest accompanied by stabilization of some labile mRNA by SGs and/or PBs construction seems to be common among responses to these drugs and other cellular stresses such as reactive oxidant intermediates and heat shock [46]. The attenuated FOS mRNA elevation following translation arrest may imply aberrant stress response of MDS-derived granulocytes.

Although both transcriptional and post-transcriptional upregulations of FOS were involved in FOS mRNA elevation following translational arrest in granulocytes, only transcriptional enhancement of FOS accounted for p38 MAPK. The p38-independency of FOS mRNA stabilization by emetine suggested the involvement of ARE-binding proteins other than KH-type splicing regulatory protein (KSRP) and tristetraprolin (TTP). KSRP and TTP, ARE-binding proteins that destabilize mRNA, were shown to be phosphorylated via MAPK p38 in response to proinflammatory cytokine stimuli, which triggered their dissociation from mRNA, resulting in stabilization of the target mRNA [47].

It is likely that HuR stabilizes FOS mRNA in emetine-treated cells, whereas the possibility of involvement of other mRNA-stabilizing factors is not excluded. Emetine increased the specific binding of HuR to FOS mRNA 3′UTR, and the FOS mRNA stabilizing effects of emetine were attenuated by suppression of HuR expression. In contrast, in the absence of emetine, the reduction of HuR did not change the decay rate of FOS mRNA, suggesting that the rapid FOS mRNA degradation in unstimulated cells is regulated by other ARE-binding proteins that destabilize target mRNA or miRNAs.

The aberrant FOS mRNA elevation by emetine in MDS granulocytes was due to insufficient stabilization of FOS mRNA but not impaired transcriptional enhancement, as MDS-derived cells showed no difference in the increase of nascent FOS mRNA and significantly faster decay of FOS mRNA in the presence of emetine compared to control cells. The insufficient stabilization of FOS mRNA in the presence of emetine can result from defects in FOS mRNA stabilizing machinery itself or acceleration of FOS mRNA degradation activity. In the latter case, FOS mRNA decay in quiescent cells could be faster in MDS than in controls. In the absence of emetine, the differences in the remaining FOS mRNA levels between MDS and controls were not statistically significant at any time points examined. If FOS mRNA is maximally degraded in unstimultaed cells, excessive FOS mRNA destabilizing activity might fail to further accelerate FOS mRNA decay.

Since no mutations were detected in 3′UTR of FOS mRNA from the patients and the reduction of HuR protein level to 30% by siRNA resulted in attenuated FOS mRNA stability in the presence of emetine, we thought that reduced expression of HuR could cause impaired mRNA stabilization in MDS patients. However, HuR protein level was decreased to less than half only in one patient. The relevance of reduced HuR expression to insufficient FOS mRNA stabilization in this patient would be confirmed by a decreased ratio of HuR-bound FOS mRNA and impaired stabilization of other HuR targets, which we were not able to analyze because the patient's condition didn't allow further study.

In most patients, impaired FOS mRNA elevation by emetine was not attributable to aberrant HuR expression. What caused the impairment of emetine-induced FOS mRNA stabilization in those patients? Even in the presence of normal level of HuR, its function can be impaired by aberrant expressions or mutations in HuR itself [48] or HuR-regulatory molecules such as transport machinery [49] and enzymes involved in phosphorylation/methylation of HuR [48]. Acceleration of FOS mRNA decay activity is conferred by overexpression of RNA-destabilizing proteins [50], [51] or miRNA [52], which may interfere with emetine-induced RNA stabilization.

In this study, we have demonstrated the impairment of stress-induced FOS mRNA stabilization in MDS, which, to our knowledge, has not been reported in hematopoietic diseases before. The mechanism of the impairment, which needs to be further studied, may provide a new insight to unveil the pathophysiology behind qualitative abnormalities in MDS granulocytes and guide rational drug design and other therapeutic interventions.
